# Reorganization of neuronal circuits of the central olfactory system during postprandial sleep

**DOI:** 10.3389/fncir.2013.00132

**Published:** 2013-08-14

**Authors:** Masahiro Yamaguchi, Hiroyuki Manabe, Koshi Murata, Kensaku Mori

**Affiliations:** ^1^Department of Physiology, Graduate School of Medicine, The University of TokyoTokyo, Japan; ^2^Japan Science and Technology Agency, CRESTTokyo, Japan

**Keywords:** olfactory bulb, olfactory cortex, adult neurogenesis, cell elimination, behavioral state, slow-wave sleep, sharp waves, sensory experience

## Abstract

Plastic changes in neuronal circuits often occur in association with specific behavioral states. In this review, we focus on an emerging view that neuronal circuits in the olfactory system are reorganized along the wake-sleep cycle. Olfaction is crucial to sustaining the animals' life, and odor-guided behaviors have to be newly acquired or updated to successfully cope with a changing odor world. It is therefore likely that neuronal circuits in the olfactory system are highly plastic and undergo repeated reorganization in daily life. A remarkably plastic feature of the olfactory system is that newly generated neurons are continually integrated into neuronal circuits of the olfactory bulb (OB) throughout life. New neurons in the OB undergo an extensive selection process, during which many are eliminated by apoptosis for the fine tuning of neuronal circuits. The life and death decision of new neurons occurs extensively during a short time window of sleep after food consumption (postprandial sleep), a typical daily olfactory behavior. We review recent studies that explain how olfactory information is transferred between the OB and the olfactory cortex (OC) along the course of the wake-sleep cycle. Olfactory sensory input is effectively transferred from the OB to the OC during waking, while synchronized top-down inputs from the OC to the OB are promoted during the slow-wave sleep. We discuss possible neuronal circuit mechanisms for the selection of new neurons in the OB, which involves the encoding of olfactory sensory inputs and memory trace formation during waking and internally generated activities in the OC and OB during subsequent sleep. The plastic changes in the OB and OC are well coordinated along the course of olfactory behavior during wakefulness and postbehavioral rest and sleep. We therefore propose that the olfactory system provides an excellent model in which to understand behavioral state-dependent plastic mechanisms of the neuronal circuits in the brain.

## Introduction

We sleep every night after experiencing a variety of events and happenings during the day. Recent studies on neuronal activities during sleep have begun to elucidate the adaptive value of postbehavioral sleep. For example, accumulating evidence has shown that the hippocampus and neocortex actively undertake the reorganization of their neural circuitry during postbehavioral sleep (Buzsaki, 1989; Diekelmann and Born, 2010). In the rodent hippocampus, synchronized activities of CA1 pyramidal cells occur in association with sharp wave (SPW)/ripple events during postbehavioral rest and sleep periods (Buzsaki, 1989). These self-organized activities during rest and sleep represent the replay of place cell activities in the CA1 region based on memory traces stored in the CA3–CA1 regions of the hippocampus during the preceding behavioral stage (Wilson and McNaughton, 1994; Lee and Wilson, 2002; Foster and Wilson, 2006). The SPW/ripple-associated replay activities of hippocampal neurons are thought to play an important role in spatial and episodic memory consolidation and concomitant reorganization of neuronal circuitry not only in the hippocampus but also in the neocortex, the target of hippocampal SPW/ripple activities (Buzsaki, 1989; Hasselmo, 1999).

Recent studies have shown that neuronal circuitries in the central olfactory system are reorganized during postbehavioral rest and sleep (Yokoyama et al., 2011). Here, we review accumulating evidence that support the idea that neuronal circuitries of the olfactory cortex (OC) and olfactory bulb (OB) undergo substantial reorganization during the rest and sleep periods subsequent to eating. In particular, we focus on the reorganization of the OB circuitry that involves incorporation or elimination of newly generated adult-born neurons into or from the preexisting neuronal circuitry in the OB.

Neuronal circuitry in the rodent central olfactory system mediates a vast variety of odor-guided behavioral responses, including approaching behaviors to the odor of foods or to the odor (or pheromone) of partners, and flight behaviors to predator odors (Doty, 1986). Although innately determined neuronal circuits mediate some of the basic odor-induced behaviors, including freezing response to the fox odor trimethylthiazoline (TMT) (Morrow et al., 2000; Kobayakawa et al., 2007), a majority of odor-guided behaviors are heavily dependent on previous experience of the odor and memory of the odor objects or odor environments. It is therefore likely that neuronal circuits in the central olfactory system are highly plastic, and reorganized on a daily basis according to the experiences of odor-guided behaviors and their consequences with the aim of improving behavioral responses to the ever-changing external odor world. It is also likely that a culminating feature of the high plasticity of the olfactory neuronal circuitry is that new neurons are continually integrated into the circuitry throughout life (Lledo et al., 2006).

Because knowledge of structure is prerequisite to an understanding of function (Crick and Koch, 2005), we first summarize the structural features of neuronal circuits in the olfactory system. We then discuss the life and death decision of adult-born new neurons along the course of the wake-sleep cycle and possible underlying mechanisms. The olfactory system consists of two parallel olfactory pathways, the main olfactory system and accessory olfactory system; in this review we focus on the main olfactory system.

## Neuronal circuitry of the main olfactory system

Olfaction is mediated by odor molecules, small volatile compounds with a molecular weight of around 25–300 daltons. To enable reception of a huge variety of odor molecules, the rodent olfactory system has developed ~1,000 types of odorant receptors (ORs), each encoded by distinct gene (Buck and Axel, 1991). ORs are G-protein-coupled seven-transmembrane proteins expressed on the cilial surface membrane of olfactory sensory neurons (OSNs) in the olfactory epithelium (OE). Individual ORs respond to a range of odor molecules that share specific molecular features (Malnic et al., 1999). Individual odor molecules bind to and are received by a specific combination of ORs.

**Figure 1:**
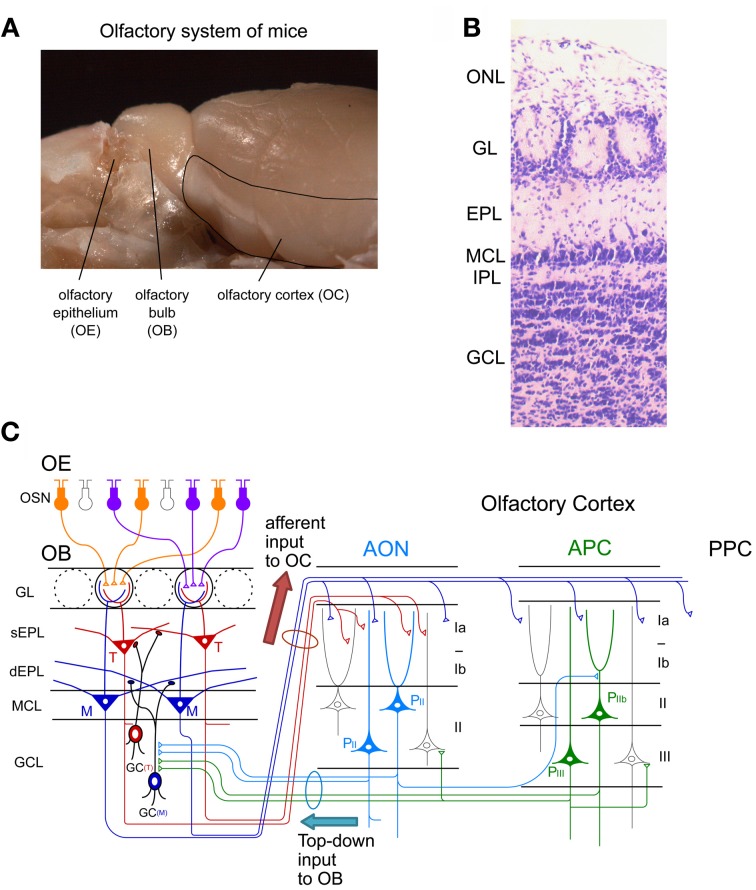
**Neuronal circuit of the olfactory system. (A)** A picture of the mouse olfactory system. The olfactory epithelium (OE), olfactory bulb (OB), and olfactory cortex (OC) are shown. **(B)** Layer structure of the OB. A nissl-stained coronal section is shown. ONL, olfactory nerve layer; GL, glomerular layer; EPL, external plexiform layer; MCL, mitral cell layer; IPL, internal plexiform layer; GCL, granule cell layer. **(C)** Schematic diagram of the neuronal circuit of the olfactory system. Connectivity of olfactory sensory neurons (OSNs) in the OE, projection neurons and local interneurons in the OB, and pyramidal cells in the anterior olfactory nucleus (AON) and anterior piriform cortex (APC) are shown. sEPL, superficial EPL; dEPL, deep EPL; M, mitral cell; T, tufted cell; GC(T), tufted cell-targeted GC; GC(M), mitral cell-targeted GC; P_II_, pyramidal cell in layer II of the AON; P_IIb_, pyramidal cell in layer IIb of the APC; P_III_, pyramidal cell in layer III of the APC; PPC, posterior piriform cortex.

Individual OSNs in the OE express a single type of OR (called “one cell-one receptor rule”) (Chess et al., 1994). Each OSN projects a single axon to a single glomerulus at the surface of the OB, the first relay center in the central olfactory system (Figure 1). Each OB contains about 1,800 glomeruli in mice. OSNs expressing a given OR project and converge their axons to a few topographically fixed glomeruli (“glomerular convergence rule”). Because of this axonal convergence of OSNs, individual glomeruli represent a single type of OR (“one glomerulus-one receptor rule”). Accordingly, the spatial arrangement of glomeruli in the OB can be viewed as a sensory map which represents numerous types of ORs (Mori et al., 2006; Mori and Sakano, 2011). It should be noted that OSNs are continuously turning over. Despite this, however, the “one glomerulus-one receptor rule” is maintained throughout life in rodents (Gogos et al., 2000).

Within glomeruli, OSN axons form excitatory synaptic connections on primary dendrites of mitral and tufted cells, the glutamatergic projection neurons in the OB (Figure 1C) (Mori, 1987; Shepherd et al., 2004). Each mitral/tufted cell projects a single primary dendrite to a single glomerulus, and thus each glomerulus and its associated mitral and tufted cells form a structural and functional module which represents a single OR. Cell bodies of mitral cells are aligned in the mitral cell layer (MCL), while those of tufted cells distribute in the external plexiform layer (EPL) (Figures 1B,C). A single glomerulus is estimated to receive primary dendrites from several tens of mitral and tufted cells (Allison and Warwick, 1949).

Mitral and tufted cells project their axons to the OC and make excitatory synapses with apical dendrites of pyramidal cells in the OC (Figure 1C). The OC is divided into several areas (Neville and Haberly, 2004). The largest of these is the piriform cortex, which is further subdivided into the anterior piriform cortex (APC) and posterior piriform cortex (PPC). In addition, the OC includes small areas of the olfactory peduncle (anterior olfactory nucleus (AON), tenia tecta and dorsal peduncular cortex), olfactory tubercle, cortical amygdaloid nuclei, lateral entorhinal cortex, and agranular insula.

Tufted cells project axons to focal targets within the rostral areas, including the AON, tenia tecta, rostrolateral part of the olfactory tubercle, and rostroventral part of the APC (Nagayama et al., 2010; Igarashi et al., 2012). In striking contrast, individual mitral cells project axons in a dispersed manner to nearly all areas of the OC (Nagayama et al., 2010; Ghosh et al., 2011; Sosulski et al., 2011; Igarashi et al., 2012). While mitral cells send signals directly to the piriform cortex, signals conveyed by tufted cells are relayed via pyramidal cells in the olfactory peduncle areas (e.g., AON) and then sent to the piriform cortex via Ib association fibers of pyramidal cells in the olfactory peduncle areas.

Each area of the OC has a pyramidal cell-based cortical structure with three distinct layers (Figure 1C). Pyramidal cells in layer IIb and III of the OC extend apical dendrites superficially into layer I and receive glutamatergic excitatory synaptic inputs from mitral/tufted cell axons (in layer Ia). Pyramidal cells give rise to association fibers that terminate in layers Ib, II and III of the same or other areas of the OC. The OC pyramidal cells send axons within the OC and outside the OC, including to the ventral agranular insular cortex, orbitofrontal cortex, amygdaloid complex, thalamus and hypothalamus (Shipley and Ennis, 1996). In addition, pyramidal cells in the AON and APC project axon collaterals massively back to the OB. The top-down centrifugal axons of the pyramidal cells distribute mostly to the GCL of the OB and terminate on inhibitory interneurons such as GCs and short axon cells (Luskin and Price, 1983; Boyd et al., 2012; Markopoulos et al., 2012).

The local neuronal circuitry in the OB is unique among cortical regions in that inhibitory interneurons outnumber excitatory projection neurons. The two most abundant populations of local interneurons in the OB are granule cells (GCs) and periglomerular cells (PGCs) (Mori, 1987; Shepherd et al., 2004). Both GCs and PGCs are continually generated even in adulthood. The number of GCs is about one order larger than that of PGCs. The total number of GCs in the adult rat OB is calculated to be around ten million, which is two orders of magnitude larger than that of mitral/tufted cells (Kaplan et al., 1985; Parrish-Aungst et al., 2007). GCs are axonless inhibitory interneurons. They have soma in the granule cell layer (GCL), and extend apical dendrites into the EPL and basal dendrites within the GCL (Figures 1B,C). Apical dendrites of GCs form dendrodendritic reciprocal synapses with lateral dendrites of mitral/tufted cells in the EPL. The dendrodendritic reciprocal synapse consists of a mitral/tufted-to-granule glutamatergic excitatory synapse and a granule-to-mitral/tufted GABAergic inhibitory synapse. A given GC makes such dendrodendritic reciprocal synapses with sister mitral/tufted cells belonging to the same glomerulus and with mitral/tufted cells belonging to different glomeruli, thereby modulating OB output through the synchronization and lateral inhibition of mitral/tufted cells (Yokoi et al., 1995; Kashiwadani et al., 1999).

A subset of GCs preferentially forms dendrodendritic synapses with mitral cells (mitral cell-targeted GCs) in the deep sublamina of the EPL (Figure 1C). Another subset of GCs preferentially forms dendrodendritic synapses with tufted cells (tufted cell-targeted GCs) in the superficial EPL. Soma of mitral cell-targeted GCs tend to distribute to the deep portion of the GCL while those of tufted cell-targeted GCs distribute to the MCL and superficial portion of the GCL.

Another type of major interneuron in the OB, PGCs, have soma in the glomerular layer (GL), and typically extend dendrites into a single glomerulus. Within the glomerulus, PGC dendrites receive excitatory inputs from the OSNs and mitral/tufted cell primary dendrites, and send output via dendrodendritic inhibitory synapses to mitral/tufted cell primary dendrites (Shepherd et al., 2004). PGCs modulate the activity of mitral/tufted cells belonging to the same glomerulus and those belonging to different glomeruli. Thus, these two types of OB local interneurons substantially modulate responses of mitral/tufted cells to olfactory sensory input. The OB also contains other types of inhibitory neurons called short axon cells, whose function has attracted recent interest but remains largely unknown (Schneider and Macrides, 1978; Eyre et al., 2008; Arenkiel et al., 2011; Boyd et al., 2012; Deshpande et al., 2013).

## Remarkable plasticity in the olfactory system: adult neurogenesis

**Figure 2:**
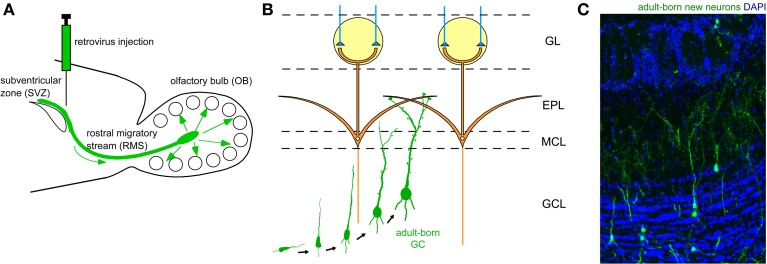
**Adult neurogenesis in the OB. (A)** Neuronal precursors are generated in the subventricular zone (SVZ) around the lateral ventricle and migrate to the OB via a specific route called the rostral migratory stream (RMS). **(B)** In the OB, neuronal precursors differentiate into GCs. Their apical dendrites form synaptic contacts with lateral dendrites of mitral cells. **(C)** Adult-born GCs are visualized by retrovirus-mediated GFP expression (green). GFP-expressing retrovirus was injected in the SVZ and the OB was analyzed 28 days after the injection. Blue, nuclear staining with DAPI.

The two major types of inhibitory interneuron in the OB, GCs and PGCs, are continually generated in the adult brain (Lledo et al., 2006). Precursors of these inhibitory interneurons are produced in the subventricular zone (SVZ) of the lateral ventricle (Figure 2A), to which ganglionic eminence- and neocortex-derived embryonic precursors for interneurons immigrate (Young et al., 2007). The newly generated neuronal precursors migrate along a specific route called the rostral migratory stream (RMS) to the OB (Figure 2A), where they mature to become GCs and PGCs (Figures 2B,C). While the production of mitral/tufted cells is limited only during the mid- to late embryonic period (Hinds, 1968; Bayer, 1983), GCs and PGCs are generated extensively during the late embryonic and early neonatal periods, with substantial production continuing in adulthood. The number of adult-born OB interneurons is very large. In rodents, at least several tens of thousands of neurons enter the OB each day (Alvarez-Buylla et al., 2001; Winner et al., 2002; Lledo et al., 2006), corresponding to roughly one percent of the total number of OB interneurons. Thus, a simple calculation suggests that adult-born interneurons will outnumber preexisting interneurons within 100 days. Although the exact percentage of adult-born interneurons in the entire interneuron population has been unclear, recent advances in molecular and developmental biology provide an estimated figure (Lagace et al., 2007; Ninkovic et al., 2007; Imayoshi et al., 2008). One report indicates that roughly 70% of GCs in the adult OB are adult-born (Imayoshi et al., 2008), suggesting that odor information processing in the adult OB is heavily dependent on adult-born GCs.

An outstanding feature of adult neurogenesis is that new neurons are synaptically integrated into preexisting neuronal circuits. Similar to embryonic- and neonatal-born GCs, adult-born GCs receive glutamatergic synaptic contact from the same two major sources, namely mitral/tufted cells via dendrodendritic synapses in the EPL and pyramidal cells in the OC via axodendritic synapses in the GCL (Figure 1C). Such synaptic incorporation of adult-born GCs occurs roughly within a month after their generation (Petreanu and Alvarez-Buylla, 2002; Carleton et al., 2003; Whitman and Greer, 2007; Kelsch et al., 2008, 2010; Katagiri et al., 2011). Axodendritic synaptic contacts from axons of OC pyramidal cells occurs earlier, at around day 14. Synaptic contacts from mitral/tufted cell dendrites become apparent later, at around day 21, and by day 28, all synaptic structures become indistinguishable from those of preexisting mature GCs. Both types of glutamatergic synapse appear to be crucial to the proper selection of new GCs for incorporation or elimination.

## Adult-born GCs are under a selection process, and either incorporated into or eliminated from the neuronal circuitry

Although a majority of adult-born GCs initially enter into the bulbar circuitry by forming immature synaptic contacts, many of these GCs are eliminated during maturation. Under normal conditions only half of new GCs succeed in living longer than one month after generation, while the other half are eliminated by apoptosis (Petreanu and Alvarez-Buylla, 2002; Winner et al., 2002; Yamaguchi and Mori, 2005). Initial excess neurogenesis and subsequent apoptotic elimination occur in both embryonic and adult neurogenesis. This selection process during embryonic development is crucial to ensuring that the neuronal circuitry has been appropriately tuned to the provision of proper information processing (Buss et al., 2006). This process also appears crucial for adult neurogenesis. When apoptotic elimination of adult-born neurons is suppressed by a caspase inhibitor, odor discrimination ability is disturbed (Mouret et al., 2009), presumably because of the presence of inappropriately incorporated adult-born GCs. However, the cellular and molecular mechanisms by which adult-born GCs are selected to survive or die is not well understood.

**Figure 3:**
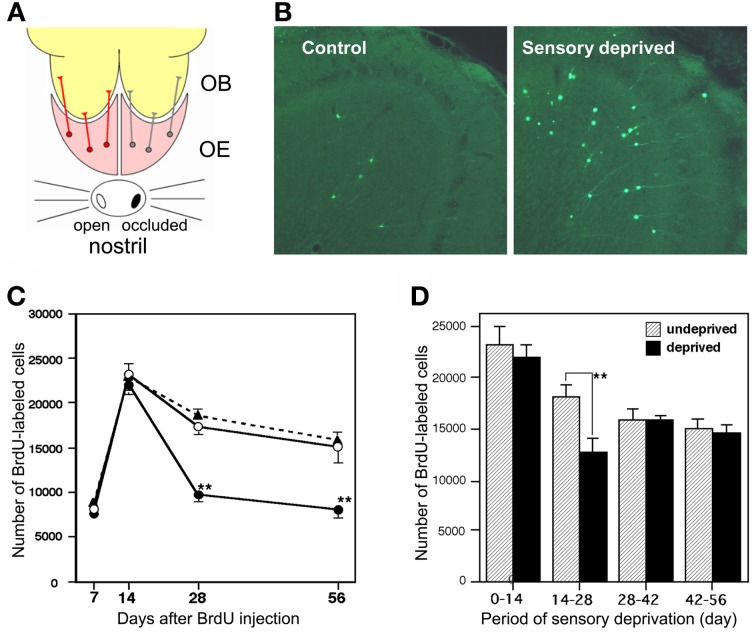
**Sensory experience-dependent life and death decision of adult-born GCs. (A)** Deprivation of olfactory sensory input by single-nostril occlusion. Olfactory sensory input is conducted from an open nostril (left) to the same side of the OE and OB, while input is not conducted to the occluded side (right) of the OE and OB. **(B)** Caspase-3-activated apoptotic GCs in the OB (green). Compared to the control (sensory input-intact) OB, a large number of apoptotic GCs are seen in the sensory-deprived OB. **(C)** Number of BrdU-labeled new GCs at various periods after labeling in the control OB (black triangles), sensory input-intact OB of mice under unilateral sensory deprivation (open circles), and sensory input-deprived OB (closed circles). Sensory deprivation remarkably decreased the number of new GCs during days 14–28 after labeling. **(D)** Effect of sensory deprivation for 14 days at various periods after BrdU labeling on the survival of BrdU-labeled new GCs. Sensory deprivation during day 14–28 decreased survival, while deprivation before or after this period showed no significant effect on survival. Modified from Yamaguchi and Mori (2005).

Survival and death of adult-born GCs depends on olfactory sensory experience. An increased survival rate of adult-born GCs was observed in those mice that were repeatedly exposed to novel odors (odor-enriched environment) (Rochefort et al., 2002). Conversely, the survival rate of adult-born GCs was decreased in anosmic mice lacking a cyclic nucleotide-gated channel in OSNs (Petreanu and Alvarez-Buylla, 2002) and in the ipsilateral OB of mice with unilateral sensory input deprivation (Corotto et al., 1994; Saghatelyan et al., 2005; Yamaguchi and Mori, 2005; Mandairon et al., 2006). GC elimination appears to be driven by an apoptotic pathway. Olfactory sensory deprivation by nostril occlusion remarkably increased apoptotic GCs, as immunohistochemically detected by the activation of caspase-3 (Figures 3A,B) (Yamaguchi and Mori, 2005).

Sensory experience-dependent plastic change in the developing central nervous system occurs during specific time windows, called critical periods. For example, ocular dominance plasticity in the primary visual cortex occurs selectively during the critical period after birth (Hensch, 2005). We examined whether there is a critical period for adult-born GCs during which their survival and death is strongly influenced by olfactory sensory experience. Newly generated GCs in adult mice were labeled by systemic BrdU injection, and the mice were then deprived of olfactory sensory input by nostril occlusion at various time periods after labeling. The results showed that sensory deprivation during days 14–28 after GC generation greatly reduced the survival of GCs, whereas deprivation before or after this period had no significant effect (Figures 3C,D) (Yamaguchi and Mori, 2005). Consistent with this observation, most apoptotic GCs showing caspase-3 activation were aged 14–28 days. These observations indicate that the sensory experience-dependent life and death decision of new GCs occurs during a critical time window at days 14–28 after their generation.

Importantly, this time window corresponds to the period when adult-born GCs make and maturate synaptic contacts with preexisting neurons (Petreanu and Alvarez-Buylla, 2002; Carleton et al., 2003; Whitman and Greer, 2007; Kelsch et al., 2008, 2010; Katagiri et al., 2011), suggesting that synaptic input plays a crucial role in the selection of adult-born GCs. Morphological examination of adult-born GCs in anosmic mice showed that young GCs destined for later elimination had already developed many synaptic structures in the EPL (Petreanu and Alvarez-Buylla, 2002). Thus, the basic strategy for GC selection appears to be that most new GCs temporally establish immature synaptic contacts with preexisting neurons before their life or death decision is made. Subsequent to the formation of immature synapses, they may undergo a “quality check” by the activity of the synapses they have made with preexisting neurons. Some are successfully incorporated for long-term function while others are eliminated by apoptosis. Although apoptotic cells are found throughout the neurogenic routes in the SVZ, RMS and OB (Biebl et al., 2000; Mandairon et al., 2003), close to 80% of apoptotic cells are found within the OB (Biebl et al., 2000). This observation further supports the notion that selection of adult-born GCs is primarily conducted at their final destination, namely neuronal circuits in the OB, where their usability might be determined by interaction with preexisting neuronal circuits.

## Selection of adult-born GCs during a behavioral state-dependent time window

Neuronal plasticity is tightly linked to the behavioral state of animals. A conspicuous alteration in behavioral state is the wake-sleep cycle. Neuronal plasticity is well organized along the wake-sleep cycle and sleep plays a critical role in the long-lasting neuronal plasticity that accompanies memory consolidation (Buzsaki, 1989; Diekelmann and Born, 2010).

Temporal incorporation and later elimination of newly generated GCs in the OB are accompanied by substantial structural reorganization of the neuronal circuitry. Because olfactory sensory experience determines the magnitude of the elimination and survival of new GCs (Corotto et al., 1994; Petreanu and Alvarez-Buylla, 2002; Rochefort et al., 2002; Saghatelyan et al., 2005; Yamaguchi and Mori, 2005; Mandairon et al., 2006), we hypothesized that structural reorganization of the OB circuitry occurs during the time course of olfactory sensory experience followed by sleep. To examine the time course of this reorganization, we thus looked for typical and appropriate behaviors of rodents that included olfactory experience and sleep.

Finding and eating foods are typical daily olfactory behaviors (Doty, 1986). Olfactory memory of a food encoded during the search and eating plays a key role in subsequent odor cue-based decision making as to the whether the animal will eat or reject the food. We therefore selected feeding behavior and subsequent sleep as a candidate behavioral sequence which accompanies structural reorganization of the OB circuitry, including the elimination of newly generated GCs.

**Figure 4:**
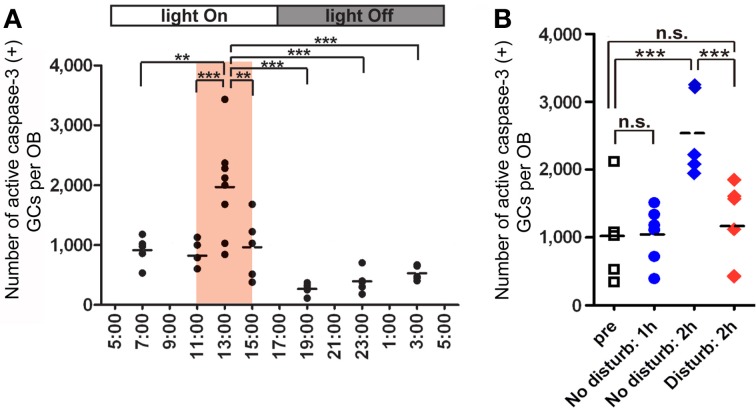
**GC elimination is promoted during the postprandial period. (A)** Apoptotic GCs increase during the feeding and postprandial periods. Mice were under restricted feeding in which food is supplied for only 4 h (11:00–15:00; an orange bar) per day. On day 10 of restricted feeding, mice were analyzed at various circadian time points for caspase-3-activated apoptotic GCs in the OB. Each dot represents the number of caspase-3-activated GCs in one animal (average of left and right OBs), and bars indicate the average number at respective time points. **(B)** Postprandial behaviors including sleep are crucial to the increase in GC elimination. After food delivery, mice were allowed to behave freely (blue) for 1 or 2 h and then analyzed for caspase-3-activated apoptotic GCs. At 1 h after food presentation (No disturb: 1h), the number of apoptotic GCs was not increased compared to just before food delivery (pre). In contrast, at 2 h after food presentation (No disturb: 2 h), the apoptotic GC number was considerably increased. When postprandial behaviors including rest, grooming and sleep were disturbed during the postprandial period (between 1 and 2 h after food delivery) (red, Disturb: 2 h), the apoptotic GC number was significantly suppressed. In **(A)** and **(B)**, each dot represents the number of caspase-3-activated GCs in one animal (average of left and right OBs), and bars indicate the average number at respective time points. ^**^, *p* < 0.01; ^***^, *p* < 0.001; n.s., not significant; One-Way ANOVA with *post-hoc* Bonferroni test. Modified from Yokoyama et al. (2011) with permission.

Under *ad libitum* feeding conditions, mice show sporadic eating behavior which is unsuitable for experimental analysis. We therefore controlled feeding behavior using a restricted feeding paradigm. Food pellets were available only during a fixed 4-h time window (11:00–15:00) (Figure 4A). After habituation to this schedule for 9 days, all mice showed extensive eating behavior followed by postprandial (after-meal) behaviors such as grooming, resting and sleeping. During the first hour of food availability (11:00–12:00), mice were mostly devoted to consuming behavior, namely food eating and water drinking. In this eating period, no increase in apoptotic GCs was observed (Figure 4B). During the subsequent hour (12:00–13:00), mice appeared to be satiated with food, and instead showed grooming, resting and sleeping. Surprisingly, the number of apoptotic GCs increased ~2-fold during this time window of postprandial behaviors (Figure 4). Most of the apoptotic GCs were newly generated GCs aged 14–28 days after generation, which corresponded to the critical period for the sensory experience-dependent survival and death decision. Perturbation of these postprandial behaviors by gently touching the mouse's body remarkably suppressed the GC apoptosis, suggesting the importance of these postprandial behaviors to the increased GC elimination.

**Figure 5:**
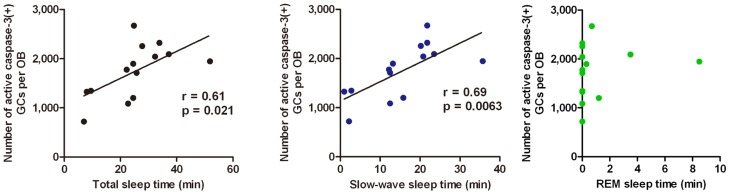
**GC elimination correlates with the occurrence of postprandial slow-wave sleep.** Behavioral state of mice during the first hour of the postprandial period is classified into waking, and the light sleep, slow-wave sleep, and REM sleep states according to neocortical EEG, neck muscle EMG and behavior. Correlation of the number of caspase-3-activated GCs with the total time length of sleep (left), slow-wave sleep (middle), and REM sleep (right) during the first hour of the postprandial period were examined. Each dot represents the data from one animal. Regression line, Pearson's *r*-value and *p*-value are indicated (left, middle). Modified from Yokoyama et al. (2011) with permission.

Sleep is the most characteristic behavior during the postprandial period and includes various stages (light sleep, slow-wave sleep and REM sleep). We then asked during which sleep stage does the increase in the number of apoptotic GCs occur. To address this question, we examined the correlation between the length of each sleep stage and the magnitude of the GC apoptosis (Figure 5). Mice showed several tens of minutes of slow-wave sleep during the first one hour of the postprandial period, and the length of the slow-wave sleep roughly correlated with the number of apoptotic GCs in the OB (Figure 5, middle). In contrast, REM sleep was rarely observed during this period, and the length of REM sleep when it did occur showed no significant correlation with the magnitude of GC apoptosis (Figure 5, right). These observations suggest that postprandial slow-wave sleep plays an important role in promoting GC elimination. It should be emphasized that the length of slow-wave sleep during the first one hour of the postprandial period is only 10–30 min in total, indicating that short time periods of slow-wave sleep in the range of a “nap” can nevertheless strongly promote GC elimination.

Does the increase in GC apoptosis occur during all sleep episodes or only during postprandial sleep? Of course, food-restricted mice sleep not only during the feeding time but also without preceding feeding. We observed that GC apoptosis did not increase during sleep periods outside the feeding time (Yokoyama et al., 2011). Thus, the increased GC apoptosis is dependent not on the sleep alone, but on the combination of feeding and subsequent sleep episodes.

## Olfactory sensory experience influences the extent of GC elimination during the postprandial period

**Figure 6:**
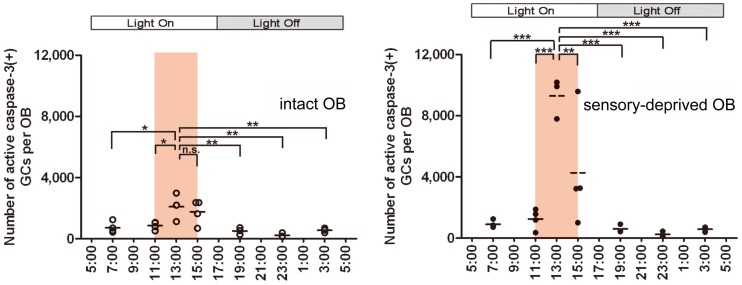
**Olfactory sensory experience influences the magnitude of GC apoptosis during the postprandial period.** In food-restricted mice (food delivery at 11:00–15:00; orange bars), olfactory sensory input was deprived unilaterally by chronic occlusion of one nostril. The number of caspase-3-activated apoptotic GCs was examined at various circadian time points in the sensory input-intact (left) and sensory input-deprived (right) OB. The number of apoptotic GCs increased dramatically in the sensory-deprived OB during the postprandial period (13:00) compared to the sensory input-intact OB. The apoptotic GC numbers just before the feeding time (11:00) and at any time period outside the feeding time were comparable between sensory-deprived and sensory input-intact OB. Each dot represents the number of caspase-3-activated GCs in one animal. Bars represent the average. ^*^, *p* < 0.05; ^**^, *p* < 0.01; ^***^, *p* < 0.001; n.s., not significant; One-Way ANOVA with *post-hoc* Bonferroni test. Modified from Yokoyama et al. (2011) with permission.

The above findings indicate that the survival and death of adult-born GCs are regulated by olfactory sensory experience. We then asked whether olfactory sensory experience influences the extent of GC elimination during the postprandial period. Mice received unilateral sensory deprivation by chronic occlusion of one nostril, and were then subjected to restricted feeding. As expected, the number of apoptotic GCs increased dramatically in the sensory-deprived OB during the postprandial period, at about 7-fold larger than that in the sensory input-intact OB of the same mice (Figure 6, 13:00). Intriguingly, the number of apoptotic GCs just before feeding time (11:00) was comparable to that in the control OB without sensory deprivation. Moreover, the number of apoptotic GCs at any period outside the feeding time did not differ from that in the OB without sensory deprivation, in spite of the fact that sensory deprivation was continuously maintained by chronic occlusion of the nostril. These results indicate that (1) olfactory sensory experience influences the extent of GC elimination during the postprandial period, and that (2) the increase in GC apoptosis in the sensory-deprived OB is restricted to a specific time window of feeding and subsequent sleep period in food-restricted mice. The sensory experience-dependent life and death decision of new GCs does not appear to be a “passive” phenomenon which is present at any behavioral period, but rather an “active” phenomenon which is tightly regulated by the sequence of olfactory sensory experience during feeding followed by postprandial sleep.

## Two-stage model for sensory experience-dependent GC selection

**Figure 7:**
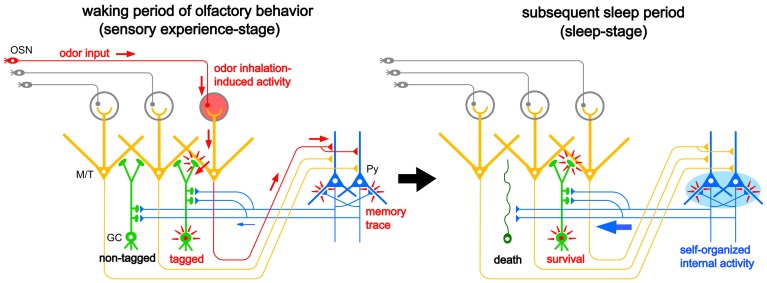
**“Two-stage model” for behavioral state-dependent GC elimination.** Adult-born GCs (green) make dendrodendritic reciprocal synapses with mitral/tufted cells (yellow, M/T) and receive top-down synaptic contacts from pyramidal cells in the OC (blue, Py). **Left panel**, during the waking period of olfactory behavior (sensory experience-stage), local sensory input from the OSNs (red arrows) activates a subset of mitral/tufted cells. Activated mitral/tufted cells activate a subset of adult-born GCs. The activated GCs might deposit “sensory experience-dependent tags” in the dendrodendritic reciprocal synapses or the cells themselves (red marks). Other adult-born GCs lacking activation by sensory experience are left “non-tagged.” Activated mitral/tufted cells further activate pyramidal cells in the OC. The memory trace of the odor experience is deposited in the association fiber synapses among pyramidal cells in the OC (red marks). **Right panel**, during the subsequent sleep period (sleep-stage), association fiber synapses among pyramidal cells in the OC are reactivated and induce synchronized firing of the pyramidal cells. This self-organized internal activity of OC pyramidal cells (a blue oval) is transferred to the adult-born GCs as synchronized top-down synaptic inputs (a thick blue arrow). The synchronized top-down synaptic inputs may contribute to the putative “reorganizing signal” that promotes GC elimination during the postbehavioral sleep period. Adult-born GCs which are tagged by sensory experience during the preceding waking period survive while adult-born GCs which are not tagged are eliminated by the “reorganizing signal.”

The majority of apoptotic GCs observed during postprandial sleep were newly generated GCs aged days 14–28 after generation, the period in which new GCs establish extensive synaptic contacts with preexisting neuronal circuitry in the OB. These observations led us to propose a “two-stage model” for the sensory experience-dependent selection of new GCs, in which the two stages represent olfactory sensory experience during food search and eating (sensory experience-stage) followed by postprandial sleep (sleep-stage) (Figure 7).

During the waking period when mice show food-searching and eating behaviors, one subset of newly generated adult-born GCs receives olfactory sensory inputs mainly via dendrodendritic synapses from mitral/tufted cells in the EPL, while the remaining subset does not (Figure 7, left). We assume that new GCs that are activated by the olfactory sensory inputs receive a kind of synaptic tagging that works as a substrate for subsequent plastic change (Frey and Morris, 1997; Redondo and Morris, 2011). These GCs may be “tagged” in the dendrodendritic synapses or the cells themselves, while other GCs that are not activated by olfactory sensory input remain “non-tagged.” Alternatively, new GCs might instead receive a “survival tag” when they receive strong synaptic input during the experience of salient olfactory-guided behaviors. New GCs that were not activated might receive a “death tag.” It is also possible that top-down axodendritic synapses from the OC pyramidal cells to new GCs are tagged by olfactory sensory experience, although whether and how a subset of new GCs receives olfactory sensory experience-dependent top-down inputs is totally unknown at present.

Importantly, although differential tagging of new GCs might occur during feeding behavior, the life and death decision of GCs is not made during feeding. During the subsequent postprandial period, food-searching and eating behaviors are overtaken by postprandial behaviors, including sleeping, and the increased GC apoptosis occurs during this period. We thus hypothesized that some sort of “reorganizing signal” enters the OB during the postprandial period and promotes GC selection according to the presence or absence or type of tag that the GCs received during the preceding waking period. Adult-born GCs “survival-tagged” by sensory experience might be selected to survive by this “reorganizing signal,” whereas other “non-tagged” or “death-tagged” adult-born GCs might be eliminated by it (Figure 7, right). Thus, the fate of individual adult-born GCs might be determined by the interplay between tagging, reflecting the memory trace of sensory experience during the waking period, and the reorganizing signal that enters the OB during the subsequent sleep period. This idea of a two-stage model of GC elimination is based on the two-stage model of memory formation and consolidation in the hippocampus, which proposes that sensory input induces memory trace formation during awake learning experience and that replay of the experience occurs for neuronal circuit reorganization during subsequent sleep or rest (Buzsaki, 1989; Diekelmann and Born, 2010).

Enhanced GC elimination during the postprandial period also resembles homeostatic synaptic downscaling during sleep (Tononi and Cirelli, 2006). It has been shown in the rodent neocortex and hippocampus that behavioral state modulates synaptic strength. Synapses become potentiated and contain more synaptic proteins and AMPA receptors after waking, while they are globally depressed (downscaled) during sleep (Vyazovskiy et al., 2008; Maret et al., 2011). In the fly brain also, synaptic size or number increases during wakefulness and decreases after sleep (Bushey et al., 2011). Importantly, sleep deprivation inhibits the synaptic homeostasis.

It is not clear whether behavioral states modulate the strength of dendrodendritic synapses on new GCs in the EPL and the top-down centrifugal fiber synapses on new GCs in the GCL. Based on the finding that GC elimination is enhanced during postprandial sleep, we speculate that the strength of these synapses on new GCs is under the modulation of behavioral state. One possibility is that new GCs lacking a net increase in total synaptic strength during feeding behavior might be eliminated by apoptosis during subsequent sleep. Sensory experience-dependent elimination of adult-born GCs during the postprandial period downscales the GC number. Because a large number of adult-born GCs are recruited in the OB every day, elimination of adult-born GCs is necessary to maintaining the overall number of GCs in the entire OB within an appropriate range. This downscaling may increase the ratio of useful vs. useless GCs, thereby improving the signal-to-noise ratio for olfactory information processing, and may make room for a successive cohort of new GCs to be integrated in preparation for the next round of new olfactory experience.

## Behavioral state-dependent signal flow in the olfactory system: interaction between the OB and the OC

To investigate neural circuit mechanisms in the life and death decision of new GCs during the sequence of eating and subsequent rest/sleep, we need to understand how the central olfactory system works not only in encoding olfactory memory of food during the waking period but also in stabilizing the resulting memory traces during the subsequent rest/sleep period.

During the waking period, odor inhalation induces spike responses of mitral/tufted cells in the OB. These activated mitral/tufted cells then activate GCs via mitral/tufted-to-granule dendrodendritic excitatory synapses in the EPL (Figure 10, upper). Mitral/tufted cells also activate pyramidal cells of the AON and APC via axons that terminate in layer Ia. Activated pyramidal cells of the AON send signals via Ib associational fibers to APC pyramidal cells. During waking states, therefore, odor information is conveyed by pathways consisting of OSNs-mitral/tufted cells-pyramidal cells in the AON and APC (Figure 10, upper). During the slow-wave sleep state, however, the OC is isolated from the external odor world by sensory gating mechanisms that diminish signal transmission from the OB to the OC (Figure 10, lower) (Murakami et al., 2005; Manabe et al., unpublished). The mechanism of this behavioral state-dependent olfactory sensory gating is not yet understood.

**Figure 8:**
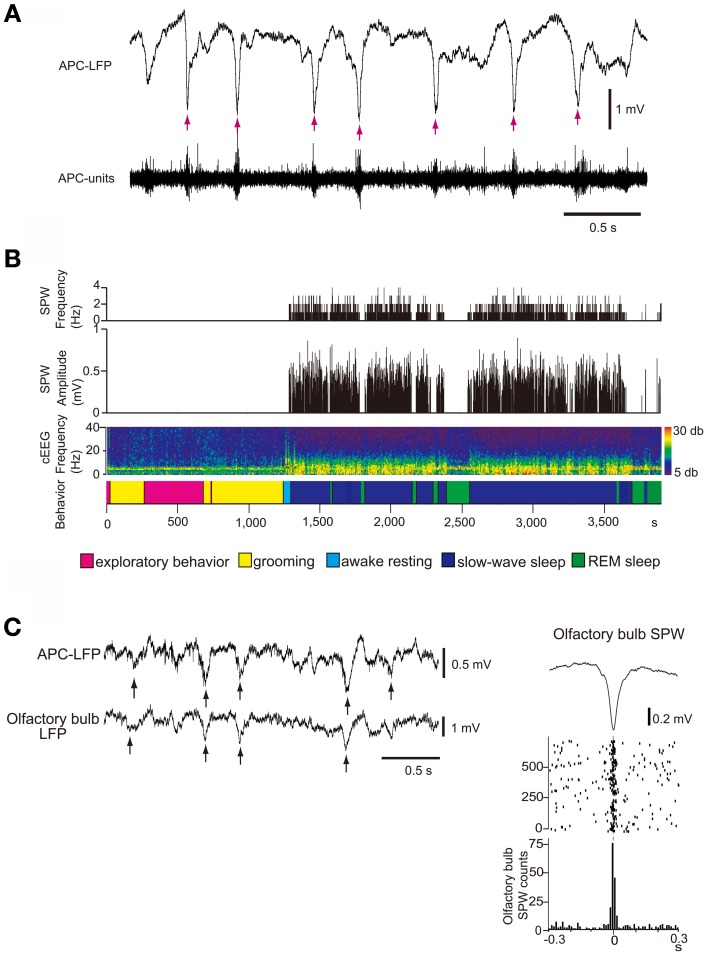
**SPW generation in the OC and its transmission to the OB during slow-wave sleep. (A)** The occurrence of OC-SPWs is associated with synchronized spike discharges of APC neurons. Simultaneous recordings of local field potentials in layer III (APC-LFP) and multiunit spike activity (APC-units) of the APC during slow-wave sleep of freely-behaving rats. OC-SPWs are indicated by red arrows. **(B)** OC-SPWs occur repeatedly during slow-wave sleep. The frequency and amplitude of OC-SPWs (top two histograms), neocortical EEG pattern (cEEG, shown by a spectrogram), and behavioral states (bottom bar). The spectrogram of the neocortical EEG chronologically represents the intensity of power (shown by color, right inset) at each frequency. The x-axis indicates time and the y-axis indicates frequency. In the bottom bar, different behavioral states are shown by distinct colors as indicated. OC-SPWs occur repeatedly during slow-wave sleep, while they are suppressed during waking and REM sleep. **(C)** OC-SPW activity is transferred to the OB as synchronized top-down inputs. Left, simultaneous recording of LFP in layer III of the APC (APC-LFP) and LFP in GCL of the OB (olfactory bulb-LFP). Arrows indicate SPWs. Right, averaged time course of olfactory bulb SPWs (top trace), and the raster plot (middle traces) and event-correlation histogram (bottom trace) of the nadir of olfactory bulb SPWs in reference to the nadir of OC-SPWs are shown. Time 0 indicates the time of nadir of OC-SPWs. Modified from Manabe et al. (2011).

What types of neural activity occur in the isolated OC during sleep? Local field potential (LFP) recording in the deep layer (layer III) of the APC during the slow-wave sleep state in freely-behaving rats revealed that the APC generates repetitive sharp negative potentials that resemble hippocampal SPWs in shape and duration (~100 ms) (Figure 8A) (Manabe et al., 2011). These sharp waves were observed in wide areas of the APC and AON and are called “olfactory cortex sharp waves” (OC-SPWs). OC-SPWs are associated with synchronized spike discharges of numerous neurons in the APC and AON (Figure 8A).

As described in the Introduction, hippocampal SPWs are thought to play an important role in memory consolidation and concomitant reorganization of the neuronal circuitry in the hippocampus and neocortex. In analogy with the function of hippocampal SPWs, we speculate that OC-SPWs might be involved in the olfactory memory consolidation and reorganization of neuronal circuitry in the central olfactory system.

**Figure 9:**
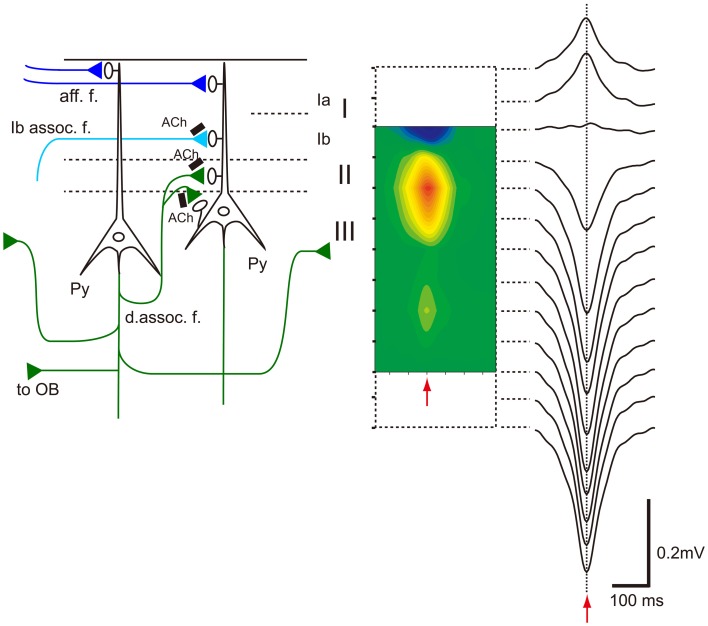
**Association fiber excitatory synapses on OC pyramidal cells participate in the generation of OC-SPWs.** Left, schematic diagram illustrating mitral cell axon (afferent fiber) excitatory synapses that terminate in layer Ia (aff. f.; dark blue lines), association fiber excitatory synapses from pyramidal cells of the AON that terminate in layer Ib (Ib assoc. f.; a light blue line), and deep recurrent association fiber (d. assoc. f.; green) excitatory synapses of APC pyramidal cells on APC pyramidal cells that terminate in layer II and III. Association fiber terminals are under the control of cholinergic presynaptic inhibition (black bars with Ach). Top-down projection from the APC pyramidal cells to the OB is also shown. Right, current source density analysis of averaged OC-SPWs in a rat under urethane anesthesia. The depth profile of averaged OC-SPW potentials is shown. Pseudo-color representation of current sink (red) and current source (blue) is also shown. Red arrows indicate the nadir of OC-SPW. OC-SPWs generate a large current sink in layer II and the adjacent superficial part of layer III. Modified from Manabe et al. (2011).

Table 1 summarizes a comparison of the properties of hippocampal SPWs and OC-SPWs. Hippocampal SPWs are a self-organized activity originating in recurrent excitatory synaptic connections among CA3 pyramidal cells (Csicsvari et al., 2000). Transient memory traces of experienced episodes are thought to be deposited in the recurrent excitatory synaptic connections. Similarly, OC-SPWs are generated by pyramidal cells of the piriform cortex and also by neurons in the endopiriform nucleus, which is located just deep to the piriform cortex. The recurrent excitatory synaptic connections among piriform cortex pyramidal cells are thought to be involved in the generation of OC-SPWs. Current source density analysis of OC-SPWs in the APC during slow-wave sleep revealed the existence of a dense current sink in layers II and III, which are deep layers in which association fibers of APC pyramidal cells form excitatory synaptic connections on dendritic spines of APC pyramidal cells (Figure 9) (Neville and Haberly, 2004; Manabe et al., 2011). Memory traces of olfactory images of objects are thought to be encoded in plastic changes occurring in the recurrent association fiber synapses among pyramidal cells of the OC (Haberly, 2001; Neville and Haberly, 2004; Wilson, 2010; Wilson and Sullivan, 2011).

**Table 1:** **Comparison between hippocampal sharp waves and olfactory cortex sharp waves**.

	**Hippocampal-SPWs**	**Olfactory cortex (OC)-SPWs**
Origin	CA3 pyramidal cells (recurrent excitation path)	Piriform cortex pyramidal cells
		Endopiriform nucleus (recurrent excitation path)
Synchronization	CA1 pyramical cells	Piriform cortex pyramical cells
Replay/reactivation	Place cell activity during exploratory behavior	Not known
Target	Hippocampus, Subiculum, Entorhinal cortex, Neocortex	Piriform cortex, Olfactory bulb, Orbitofrontal cortex, Cortical amygdaloid nuclei, Olfactory tubercle
Behavioral states	Awake resting Slow-wave sleep	Slow-wave sleep
Possible function	Consolicdation of spatial and episodic memory	Not known
Abnormal activity	Hippocampal epilepsy	The area tempestas

While hippocampal SPWs are associated with synchronized discharges of CA1 pyramidal cells, OC-SPWs accompany synchronized discharges of piriform cortex pyramidal cells. Replay of CA1 place cell ensemble activity occurs during the short time window of individual hippocampal SPWs. However, it is not known whether replay or reactivation of OC ensemble activity occurs during OC-SPWs. Hippocampal SPWs travel from the CA1 region, through the subiculum, entorhinal cortex and up to the wide areas of the neocortex. Similarly, OC-SPWs travel to all parts of the piriform cortex, AON, OB, cortical amygdaloid nuclei, olfactory tubercle and orbitofrontal cortex.

Both hippocampal SPWs and OC-SPWs occur in a behavioral state-dependent manner. They are absent during awake exploratory behavior and REM sleep. Hippocampal SPWs occur selectively during slow-wave sleep, awake resting and consuming behavior. OC-SPWs occur during slow-wave sleep, and SPW-like potentials are present in the APC during postprandial rest (Figure 8B) ((Manabe et al., 2011); Komano-Inoue et al., unpublished).

**Figure 10:**
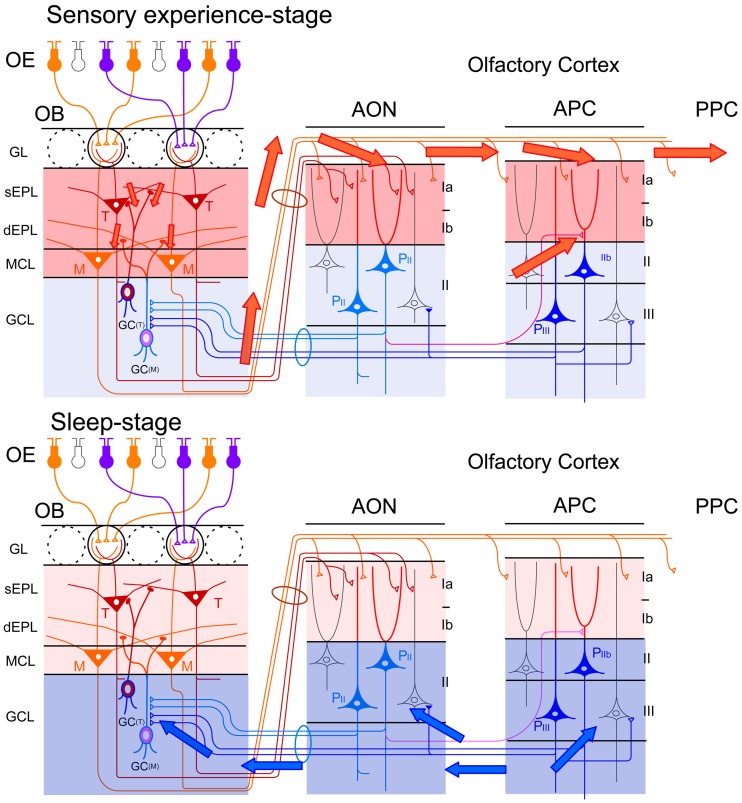
**Behavioral state-dependent signal flow between the OB and OC. Upper panel**, during the waking period (sensory experience-stage), information about the external odor world is efficiently transferred from mitral (M) and tufted (T) cells in the OB to pyramidal cells (P) in the AON, APC and PPC (red arrows). Olfactory sensory inputs from the OE to the OB activate synapses in the sEPL, dEPL and MCL of the OB. Outputs from mitral and tufted cells in the OB to the OC activate synapses in the layer Ia of the AON and APC. Activated pyramidal cells in the AON activate synapses in the layer Ib of the APC via their association fibers (a red line in the deep layer). Layers with activated synapses are highlighted with red. Synaptic inputs of recurrent association fibers to pyramidal cells are reduced by cholinergic presynaptic suppression. **Lower panel**, during the slow-wave sleep period (sleep-stage), afferent fiber inputs to the OC are blocked by behavioral state-dependent sensory gating. In contrast, synaptic inputs of recurrent association fibers to pyramidal cells in layer II and III of the olfactory cortex are released from cholinergic presynaptic inhibition and internally generate synchronized spike discharges of pyramidal cells in the AON, APC and PPC. The synchronized spike discharges of pyramidal cells in the OC travel back to GCs in the GCL of the OB as synchronized top-down synaptic inputs (blue arrows). Layers with activated synapses are highlighted with blue.

Given that a large population of pyramidal cells in the AON and APC send massive top-down centrifugal fibers to GCs in the OB (Luskin and Price, 1983), the OC-SPW-associated synchronized discharges of numerous AON and APC neurons would likely cause massive synchronous excitatory synaptic inputs to GCs. Indeed, simultaneous recording of LFP in the ipsilateral APC layer III and OB GCL showed that SPW-like potentials in the OB occurred in close temporal proximity to OC-SPWs (Figure 8C). Synchronous discharge of APC neurons occurs repeatedly during slow-wave sleep and causes repeated strong and synchronized synaptic excitation of GCs in the OB (Figure 10, lower).

OC-SPWs occur in the absence of olfactory sensory inputs, suggesting that they are a self-organized activity originating in the piriform cortex. During awake states, recurrent association fiber synapses on APC pyramidal cells are presynaptically inhibited by tonic cholinergic tone (Hasselmo and Bower, 1992). During slow-wave sleep, however, reduced cholinergic tone liberates the recurrent association fiber from this cholinergic suppression to promote the generation of highly synchronized discharges of pyramidal cells and OC-SPWs (Figures 9, 10).

## Possible mechanisms underlying the behavioral state-dependent selection of adult-born GCs

**Figure 11:**
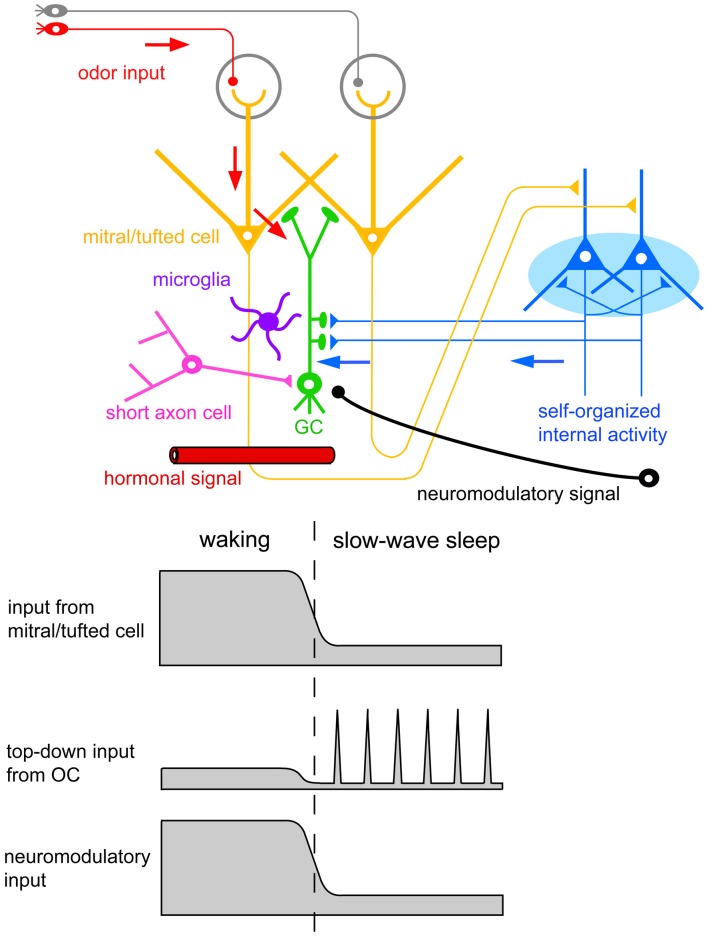
**Possible contributors to the behavioral state-dependent life and death decision of adult-born GCs.** Adult-born GCs receive excitatory synaptic inputs from mitral/tufted cells via dendrodendritic synapses (red arrows). These inputs represent olfactory sensory inputs and are strongly activated during waking olfactory behavior. Adult-born GCs also receive excitatory synaptic inputs from pyramidal cells in the OC via axodendritic synapses (blue arrows). These inputs represent self-organized internal activity of OC pyramidal cells and are strongly activated during the subsequent slow-wave sleep period. Adult-born GCs receive neuromodulatory signals including noradrenergic, cholinergic, and serotonergic inputs. These neuromodulatory inputs are elevated during waking olfactory behavior and suppressed during the subsequent slow-wave sleep period. Behavioral state-dependent hormonal signals may be also involved in the life and death decision of adult-born GCs. Deep short axon cells in the OB form GABAergic synapses onto adult-born GCs. Top-down inputs from OC pyramidal cells may induce inhibitory synaptic inputs on adult-born GCs via the activation of deep short axon cells. Resident microglia in the OB play a crucial role in sensory experience-dependent GC elimination. Behavioral state-dependent life and death decision of adult-born GCs may operate with the cooperation of these signal systems and cell types.

The finding of increased GC death during postprandial sleep raises a number of interesting questions. One major question is what kind of signals new GCs receive during the sequence of eating behavior and postprandial sleep. A second is how these signals relate to the life and death decision of new GCs during sleep. One possible scenarios is that (1) new GCs receive odor-induced signals from mitral and tufted cells during eating, but repetitively receive OC-SPW-associated synchronized top-down inputs from the OC during subsequent sleep; and that (2) the synchronized top-down inputs are crucial in promoting GC elimination (Figures 7, 10, 11).

In this scenario, olfactory sensory input is transmitted from mitral/tufted cells to new GCs via the dendrodendritic synapses in the EPL. Some GCs might be “tagged” by the synaptic inputs while others are left “non-tagged.” In spite of the tagging, the life and death decision of new GCs is not conducted during waking states. Rather, only during the subsequent sleep period do OC neurons generate memory trace-based synchronized activity (OC-SPWs) and send synchronized top-down inputs to new GCs. This signal may trigger mechanisms that eliminate “non-tagged” GCs while promoting long-lasting incorporation of “tagged” GCs.

We are currently examining this hypothesis, and have observed that pharmacological suppression of the synchronized top-down inputs during the postprandial period in freely-behaving mice inhibits the increased GC apoptosis (Komano-Inoue et al., unpublished observation). This observation favors the notion that the major contributor to the putative “reorganizing signal” that promotes GC elimination during the postprandial period is the synchronized top-down input from the OC to the OB (Figures 7, 10, 11).

Enhanced GC death occurs during postprandial sleep but not during sleep without preceding eating. In contrast, OC-SPW-associated synchronized top-down inputs to GCs always occur during slow-wave sleep regardless of the presence or absence of preceding eating. Thus, the synchronized top-down inputs from the OC alone may not be sufficient to trigger GC elimination. Deposition of putative tag signals during preceding waking may be prerequisite, and the life and death decision of new GCs might be determined after collation of the top-down reorganization signal with the putative deposited tag signals. Further, other behavioral state-dependent signals such as neuromodulatory and hormonal signals might also be involved in triggering GC elimination.

A key question yet to be answered is how neuromodulatory inputs to the OB influence GC elimination during postprandial sleep. The OB is targeted by subcortical neuromodulatory systems (Shipley and Ennis, 1996) that include cholinergic input from the horizontal limb of the diagonal band of Broca, noradrenergic input from the locus ceruleus, and serotonergic input from the raphe nuclei (Figure 11). Olfactory sensory experience during feeding and mating strongly increase noradrenergic signals (Brennan et al., 1990; Wellman, 2000). In addition, a variety of olfactory learning depends on neuromodulatory signals to the OB. Blockade of noradrenergic (Sullivan et al., 1989; Veyrac et al., 2009) or cholinergic signals (Devore et al., 2012) in the OB perturbs olfactory learning.

Female mice form olfactory recognition memory to male mouse pheromones at mating. The memory trace for this is deposited as the plastic change in the dendrodendritic reciprocal synapses between mitral cells and GCs in the accessory OB (Kaba and Nakanishi, 1995). The mating-induced increase in noradrenalin reduces granule-to-mitral dendrodendritic inhibitory synaptic transmission and induces olfactory memory of male pheromones. It is possible that an increase in neuromodulatory tone during olfactory learning may be important to the formation of memory traces in the dendrodendritic synapses, which can be used later in the life and death decision of new GCs. In the main OB, noradrenergic fibers from the locus ceruleus are distributed predominantly in the GCL (McLean et al., 1989), and GCs express several subtypes of adrenergic receptors (McCune et al., 1993; Nai et al., 2010).

Cholinergic fibers primarily innervate the GL and GCL in the OB (Kasa et al., 1995), and GCs express several subtypes of cholinergic receptors (Le Jeune et al., 1995). During the waking period, enhanced cholinergic tone reduces granule-to-mitral dendrodendritic synaptic transmission by a presynaptic inhibition mechanism (Tsuno et al., 2008). The top-down synaptic inputs from the OC to GCs are also reduced during the waking period, presumably by cholinergic presynaptic inhibition (Manabe et al., 2011). In the absence of cholinergic tone during slow-wave sleep, both the granule-to-mitral synaptic transmission and top-down synaptic inputs are enhanced as a result of the release from cholinergic presynaptic inhibition. These results suggest the involvement of a behavioral state-dependent change in neuromodulatory inputs in the regulation of GC death during postprandial sleep. In fact, the effects of systemic modulation of noradrenergic or cholinergic signals on the survival of new GCs have been well documented (Cooper-Kuhn et al., 2004; Kaneko et al., 2006; Veyrac et al., 2009; Moreno et al., 2012). We thus assume that the integration of odor-induced glutamatergic signals that occur during odor experiences, synchronized top-down glutamatergic inputs during sleep, and behavioral state-dependent changes in neuromodulatory signals is the key mechanism for sensory experience-dependent and behavioral state-dependent GC selection (Figure 11). Further study is necessary to examine how the neuromodulatory signals contribute to the life and death decision of new GCs.

Hormonal signals also depend on behavioral state and substantially influence olfactory neurogenesis (Figure 11). An increase in prolactin secretion by sexual and social behaviors promotes cell proliferation in the SVZ (Shingo et al., 2003; Mak et al., 2007; Mak and Weiss, 2010). Food restriction and food intake recruit a variety of stress- and energy status-related hormonal signals (Dallman et al., 1993; Gao and Horvath, 2007). Stress-induced glucocorticoid decreases cell proliferation in the SVZ (Lau et al., 2007). Although little is known at present, the life and death decision of new GCs might be regulated by a behavioral state-dependent change in hormonal signals.

Other potential contributors to the life and death decision of new GCs are deep short-axon cells and microglia in the OB (Figure 11). In addition to glutamatergic inputs, new GCs receive GABAergic synaptic inputs from deep short-axon cells in the OB (Arenkiel et al., 2011; Deshpande et al., 2013). Because deep short-axon cells receive direct top-down synaptic inputs from the OC (Boyd et al., 2012), the top-down inputs may activate inhibitory synaptic inputs onto new GCs via deep short-axon cells, in addition to direct excitatory synaptic inputs. Microglial cells are present at high density in the OB and contribute to GC elimination. Activation of microglial cells is crucial to the enhanced GC elimination by sensory deprivation (Lazarini et al., 2012). An understanding of how these signal systems and cell types work together in promoting the behavioral state-dependent life and death decision of new GCs is therefore important.

## Significance of behavioral state-dependent selection of adult-born GCs

Why is GC death enhanced during sleep? Cell death is an irreversible process: once the apoptotic machinery is initiated, the cell is destined to be eliminated from the circuit. This suggests that the cell death process of new GCs occurs under strict regulation during slow-wave sleep, unperturbed by unpredictable olfactory sensory inputs. Furthermore, if elimination or incorporation of new neurons were to occur during awake behavior states, this would cause severe disturbance in odor information processing. Therefore, neuronal circuits in the olfactory system require that the processing of external odor information and reorganization of connectivity occur during different time windows. These ideas are in accord with the notion that the brain requires separate time windows (awake and sleep periods) for the active processing of external sensory information and the structural reorganization of its neuronal circuitry.

Refinement of OB circuits by the elimination of “non-tagged” or “death-tagged” GCs during sleep may increase the signal-to-noise ratio for odor information processing, as proposed for synaptic downscaling during sleep (Tononi and Cirelli, 2006). When animals wake up, their odor information processing ability might be improved such that odor-cued behaviors can be more efficiently executed. In addition, elimination of GCs may make room for the integration of successive cohorts of new GCs during subsequent wake-sleep cycles. We observed that enhanced elimination of preexisting GCs in a local area of the OB by local injection of immunotoxin facilitates the incorporation of newly generated GCs in the local OB area (Murata et al., 2011). This observation supports the idea that elimination of less useful GCs during sleep would facilitate the incorporation of new useful GCs during subsequent wake-sleep cycles. By using and repeating the wake-sleep cycle as a single unit for olfactory experience-based circuit reorganization, the olfactory neuronal circuit may be continually remodeled to meet the ever-changing odor circumstances.

It has been demonstrated that odor learning, but not simple odor experience, is important for the survival of new GCs (Alonso et al., 2006; Mouret et al., 2008, 2009; Sultan et al., 2010). We speculate that food finding and eating periods provide rich opportunity for odor-food association learning with the animal's decision making to eat or reject the encountered food. This notion is in agreement with the observation that the life and death decision of new GCs is promoted during postprandial sleep but not during sleep without preceding eating. Beside the odor-food association learning during the eating period, mice and rats show olfactory learning in a variety of occasions, including mating, encountering danger (odor-danger association learning), and social behaviors (Keverne, 2004; Sanchez-Andrade and Kendrick, 2009; Landers and Sullivan, 2012). It would be interesting to examine which types of olfactory learning induce enhanced GC death during postbehavioral sleep.

## Coordination of plastic change in the OB, OC and other brain regions

Odor learning-induced plastic changes occur not only in the OB, but widely in the central olfactory system. For example, major plastic changes associated with olfactory memory are considered to occur in association fiber synapses of pyramidal cells in the piriform cortex. The association fiber synapses are active in odor information processing during waking, causing odor-induced activation of targeted pyramidal cells in the piriform cortex (Poo and Isaacson, 2011). Association fiber synapses are highly plastic. They exhibit NMDA-dependent long-term potentiation (LTP) *in vitro* (Kanter and Haberly, 1990; Poo and Isaacson, 2007), and synaptic transmission of association fibers is enhanced by associative odor learning in rats (Saar et al., 2002). We speculate that deposited memory traces in the association fiber synapses might be consolidated by the reactivation of synaptic connections during the subsequent sleep period, whose activity is represented as OC-SPWs. In fact, firing activity of piriform cortical neurons during the slow-wave state is influenced by odor stimulation during the preceding fast-wave state (Wilson, 2010).

Because OC-SPW-associated activity during slow-wave sleep presumably depends on the plastic changes in association fiber synapses, top-down inputs from the OC to the OB reflect olfactory memory information stored during the awake behavior period. An intriguing possibility is that the top-down synaptic inputs are not random but rather targeted to a selective population of GCs. Only the targeted GCs might be plastically modulated by the top-down inputs. Furthermore, the synaptic efficacy of the top-down inputs onto GCs is plastic. LTP can be induced in the top-down synapses both *in vitro* (Gao and Strowbridge, 2009; Nissant et al., 2009) and *in vivo* (Manabe et al., 2011). Thus, plastic changes in connectivity may occur widely in a coordinated manner across the entire neuronal circuitry of the OB and OC.

The neuronal circuitry of the OB and OC functions within a large network of the central nervous system. The OC has massive reciprocal connections with the amygdaloid complex and orbitofrontal cortex (Shipley and Ennis, 1996). During odor-guided behaviors, odor information is transferred from the OB to these regions via the OC. During slow-wave sleep periods, generation of OC-SPWs is under the control of slow-wave activity in the orbitofrontal cortex (Onisawa et al., unpublished). Thus, plastic change in the OC circuitry during sleep may be coordinated with the plasticity in the orbitofrontal cortex. In this context, the top-down inputs from the OC to the OB would reflect the coordinated activity of the OC and orbitofrontal cortex.

## Conclusion

Neuronal circuits in the olfactory system require highly plastic properties to acquire new odor-guided behaviors in response to the changing external odor world, and adult-born GCs in the OB provide remarkable plasticity to the neuronal circuit. The survival rate of adult-born GCs is influenced by olfactory sensory experience. The sensory experience-dependent life and death decision of new GCs occurs extensively during the 2–4 weeks after GC generation, and during this period new GCs make synaptic contacts with the preexisting neuronal circuit. At this critical period new GCs receive olfactory sensory inputs from mitral/tufted cells in the OB and top-down inputs from pyramidal cells in the OC. Further, the life and death decision of new GCs occurs in a behavioral state-dependent manner. Elimination of new GCs occurs extensively in the sequence of feeding behavior and postprandial slow-wave sleep. During waking, olfactory sensory information is efficiently transmitted from the OB to the OC. During slow-wave sleep, in contrast, signal transmission from the OB to the OC diminishes, and deep-association fiber-mediated synchronous firing of OC pyramidal cells occurs, which causes synchronous top-down inputs from the OC to the OB. In addition, GCs are targeted by neuromodulatory systems whose activities considerably change along the course of the wake-sleep cycle. These observations suggest the hypothesis that the key mechanism for sensory experience-dependent and behavioral state-dependent GC selection is integration across the distinct time windows of odor-induced glutamatergic input signals during odor experiences, synchronized top-down glutamatergic inputs during sleep, and behavioral state-dependent changes in neuromodulatory signals. On this basis, the GC selection process represents the coordination of a variety of activities of wide brain areas across different behavioral states. New GCs in the olfactory neuronal circuit might provide a good platform to understanding how neuronal circuits are plastically modulated in order to change animal behavioral outputs in response to the ever-changing external world.

### Conflict of interest statement

The authors declare that the research was conducted in the absence of any commercial or financial relationships that could be construed as a potential conflict of interest.
